# Impact of the National Drug Price Negotiation policy on the price, usage, and affordability of anticancer medicines in Shandong Province, China

**DOI:** 10.3389/fpubh.2024.1368718

**Published:** 2025-01-07

**Authors:** Yaqun Sun, Yan Qiang, Yongxuan Duan, Yan Song

**Affiliations:** ^1^School of Health Care Security, Shandong First Medical University & Shandong Academy of Medical Sciences, Jinan, China; ^2^Shandong Institute of Medical and Health Information, Jinan, China

**Keywords:** National Drug Price Negotiation, anticancer drugs, drug prices, affordability, China

## Abstract

**Objective:**

In order to reduce the price and increase the accessibility of innovative medicines, China has implemented the National Drug Price Negotiation (NDPN) since 2016. Anticancer drug is the largest category of NDPN and the number continue to increase. This study evaluated the impact of this policy on the price, utilization rate and affordability of anticancer drugs based on the experiences of Shandong province.

**Methods:**

25 anticancer drugs were included in this study involved the NDPN in the year 2018 and 2019. Data on prices and utilization of the policy related drugs from 2017 to 2022 were collected from Shandong Province, using an adaptation of the WHO/HAI methodology. Prices were measured as Median Price Ratio (MPR). Usage was measured as Defined Daily Doses (DDDs). Affordability was measured as days of daily per capita disposable income required for the cost of 1 month’s treatment. The Mann–Whitney U test was used to estimate the significance of the difference in the change in the MPRs before and after the negotiation.

**Results:**

The data of this study come from 42 key monitoring medical institutions in Shandong Province, including 31 tertiary medical institutions and 11 secondary medical institutions. There has been a significant reduction in the MPR following NDPN, with a median MPR of 0.57 in 2022, and the prices of anticancer medicines were generally lower than IPR. During the period from 2017 to 2022, the total usage of the 25 negotiated medicines continued to rise. With the implementation of negotiation policy, the average number of days of disposable income per capita required for 1 month of medicine costs changed from 104 days to 36 days and 256 days to 80 days for urban and rural residents, respectively. The affordable proportion of anticancer medicines is still not high.

**Conclusion:**

The NDPN policy has reduced the prices of anticancer drugs and greatly improved their affordability. More attention should be paid to improve the affordability to the rural and the poor patients. It is essential to encourage the research and development of high-quality generic drugs to strengthen reasonable market competition, as well as improve the multi-tiered medical security system.

## 1 Introduction

Cancer has become one of the major diseases endangering human health and the leading cause of death worldwide. The World Health Organization estimated in 2019 that the number of cancer cases is the primary or secondary cause of death in 112 out of 183 countries (1) and the number of cancer cases worldwide will increase to 28.4 million by 2040 (2). China’s malignant tumor incidence and mortality rates are 24 and 30% of the global rate respectively, and its age-standardized incidence and mortality rates are higher than the global average (3). Given the unprecedented rate of population aging and the increasing incidence of cancer, the demand for anticancer drugs will continue to increase in the future. Innovative drug treatments and quality medical care have improved patient prognosis to some extent. However, they also result in high medical expenses, and the burden of medication on patients has always been a serious problem. Studies have shown that medicine costs account for 70% of total medical expenditures among cancer patients receiving active treatment (4). Most cancer patients in developed and developing countries have suffered financial toxicity: subjective psychological stress and objective financial burden (5–7), and at least since 2000, cancer patients’ financial problems have escalated much faster than monetary inflation (8). Although targeted cancer drugs have good clinical benefits, their high price has raised concerns and many patients cannot afford targeted anticancer drugs (9). Since patients with malignancies require long-term treatment or even lifelong medication, the financial hardship caused by medicine expenses can last from the beginning of the active treatment period until long after treatment is completed.

Research has shown that the Chinese government has been committed to providing equitable and quality healthcare in recent years, and promoting innovative development in the domestic pharmaceutical industry (10). In order to lower drug prices, promote the availability of innovative anticancer drugs and reduce the burden on patients, the Chinese government has actively explored and formulated a National Drug Price Negotiation (NDPN) policy. That is, the medical insurance agency negotiates with representatives of relevant pharmaceutical companies to determine drug prices and payment standards so that the drugs can be included in the National Reimbursement Drug List (NRDL) at comparatively low prices. After the negotiations, the provinces should make the negotiation results public on the Internet in a timely manner. Patients can reduce their drug burden to a certain extent by purchasing drugs at the reimbursed price. To ensure the use of negotiated drugs, public hospitals must purchase drugs via the provincial drug procurement platform and the price of drugs cannot exceed the negotiated price (11). A significant motivation for pharmaceutical companies to engage in negotiations is the potential for rapid expansion in market share following the inclusion of their drugs on the national reimbursement drug list. This shift could facilitate a transition from profit maximization through high prices to a more lucrative strategy based on high sales volumes.

The policy of drug price negotiation can be traced back as early as 2009. The State Council of China launched a healthcare reform scheme in March 2009, in which it proposed the establishment of a drug negotiation mechanism to strengthen the role of medical insurance as a constraint on drug costs (12). In 2016, the National Health Commission, National Development and Reform Commission, and the Ministry of Human Resources and Social Security initiated the first-round national-level drug price and NRDL access negotiation, successfully negotiating three drugs: Tenofovir for chronic hepatitis B, Gefitinib and Icotinib for lung cancer; the prices of these three drugs were reduced by more than 50% (13). In 2017, the Ministry of Human Resources and Social Security conducted the second round of negotiation, and 36 drugs were included in the list, which included 18 anticancer drugs (15 Western drugs and 3 Chinese drugs). In 2018, the National Healthcare Security Administration (NHSA) was established and held the third round of special talks on anticancer drugs, with 17 anticancer drugs included in the catalog. Since then, the NHSA has regularly conducted national drug price negotiations, and now that the 7th negotiation has been successfully concluded, the agreement period has been enriched with anticancer medicines year by year, and new anticancer drugs represented by small-molecule targeted drugs and large-molecule monoclonal antibody drugs have been occupying a large proportion (14). The level of medicine coverage for Chinese oncology patients is gradually improving.

Most previous studies have focused on assessing the impact of the first two rounds of national drug price negotiations (15–17). One study found that the first round of negotiations reduced the prices of cancer drugs and increased hospital purchases, but only two drugs were included and there was no analysis of drug affordability (15). Another study found that the usage and affordability of anticancer drugs had increased in 11 provinces after the second round of negotiation. However, the study investigated only three drugs, and even among those, only some experienced marginal improvements in their availability (16). In addition, research in Nanjing also found that the second round of negotiation increased the utilization of 15 drugs and reduced the burden on patients. However, this study was only carried out in one city in China with a high level of economic development and medical treatment, so the results are not universal (17).

Additionally, the NHSA established in 2018, has the function of formulating rules for access to the health insurance list, organizing and implementing national drug price negotiations, and supervising the procurement of drugs (18). The impact of drug price negotiations organized in a centrally managed manner may differ from the first two rounds of negotiations. Therefore, the objective of this study was to analyze the impact of NDPN on the price, usage, and affordability of anticancer medicines negotiated in 2018 and 2019, evaluate the effectiveness of policy implementation and inform subsequent research.

## 2 Data and methods

### 2.1 Study setting

Shandong Province, located on the eastern coast of China, has a population of over 100 million, and the population aged 60 and above accounts for more than 20%. It is one of the provinces with the highest cancer prevalence rates in China. Malignant tumors such as lung cancer, gastric cancer and liver cancers are the most common cancer types in Shandong Province. Shandong province’s gross provincial product in 2022 was RMB 87,435.1 billion (18), ranking third among 31 provinces in mainland China. In order to reduce the burden of patients’ medication, Shandong Province is one of the first provinces in China to focus on the health care security of anticancer drugs. As early as 2016, Shandong began to explore provincial-level drug price negotiation, and included 18 kinds of drugs in the scope of major illness insurance payment, among which 15 kinds were anticancer drugs, accounting for a very high proportion (19). Shandong Province has always actively followed up and implemented the NDPN policy, and clearly announced that all the anticancer drugs involved in the national negotiations will be included in the medical insurance catalog (20), which effectively guaranteed the stable supply and rational use of the negotiated anticancer drugs. In addition, the Shandong Provincial Health Insurance Bureau also proposed implementing province-wide special centralized procurement of more than 100 types of commonly used anticancer drugs to reduce the prices of anticancer drugs in 2018 (21). Given its developed economy, large population, and early initiatives for anticancer medicine security, Shandong could serve as a prominent case study.

### 2.2 Drug selection

Anticancer drugs negotiated by the national health insurance in 2018 and 2019 were selected for this research. A total of 17 anticancer drugs were included in the special negotiation of anticancer drugs in 2018, and 9 new anticancer drugs were included in the 2019 national negotiation. The study identified 25 anticancer drugs (17 in 2018 and 8 in 2019) after removing proprietary Chinese drugs with no usage data. Please refer to Table 1 for a detailed list of these drugs.

**Table 1:** Information of 22 kinds of national negotiation anticancer medicines.

	Generic name	Launch time in China	Time of entry into the NRDL	Indications listed in NRDL
1	Afatinib	2017	2018.10	Non-small cell lung cancer
2	Alectinib	2018	2019.12	Non-small cell lung cancer
3	Axitinib	2015	2018.10	Renal cell carcinoma
4	Azacitidine	2018	2018.10	Myelodysplastic syndrome / chronic myelomonocytic leukemia / acute myeloid leukemia
5	Anlotinib	2018	2018.10	Non-small cell lung cancer
6	Olaparib	2018	2019.12	Recurrent epithelial ovarian cancer / fallopian tube cancer / primary peritoneal cancer
7	Octreotide	1994	2018.10	Gastrointestinal pancreatic endocrine tumor
8	Osimertinib	2017	2018.10	Liver cancer
9	Pyrotinib	2018	2019.12	breast cancer
10	Fruquintinib	2018	2019.12	Colorectal cancer
11	Crizotinib	2013	2018.10	Non-small cell lung cancer
12	Raltitrexed	2010	2019.12	Colorectal cancer
13	Ruxolitinib	2017	2019.12	Myelofibrosis
14	Nilotinib	2009	2018.10	Chronic myeloid leukemia
15	Pertuzumab	2018	2019.12	breast cancer
16	Pegaspargas	2009	2018.10	Childhood acute lymphoblastic leukemia
17	Pazopanib	2017	2018.10	Renal cell carcinoma
18	Regorafenib	2017	2018.10	Liver cancer / colorectal cancer / gastrointestinal stromal tumor
19	Ceritinib	2018	2018.10	Non-small cell lung cancer
20	Sunitinib	2007	2018.10	Renal cell carcinoma / gastrointestinal stromal tumor / pancreatic neuroendocrine tumor
21	Vemurafenib	2017	2018.10	Melanoma
22	Cetuximab	2011	2018.10	Colorectal cancer
23	Sintilimab	2018	2019.12	Hodgkin’s lymphoma
24	Ibrutinib	2017	2018.10	Mantle cell lymphoma / chronic lymphocytic leukemia / small lymphocytic lymphoma
25	Ixazomib	2018	2018.10	Multiple myeloma

### 2.3 Data collection

The data for this study is derived from the PDB Drug Comprehensive Database, which is under the supervision of the PRC Ministry of Industry and Information Technology (MIIT). The database covers a wide range of drug sales and utilization data from hospitals in major cities in China, making it the most extensive and highly authoritative database in China. As the database with the largest amount of information, the widest coverage and the most convenient use in China’s pharmaceutical industry, it includes information on drug research and development, production, sales and marketing, and has been widely and deeply applied in all kinds of researches. We extracted drug procurement data from the database of key monitoring hospitals in Shandong Province (It contains 42 medical institutions, of which 31 are tertiary medical institutions and 11 are secondary medical institutions.), including the names, manufacturers, consumption sum, consumption quantity, dosage forms, specifications and other information of 25 national anticancer drugs from 2017 to 2022. In order to facilitate statistical analysis, we used the defined daily dose (DDD) to standardize drugs with different specifications and dosage forms. The data of urban and rural per capita disposable income in Shandong Province comes from “Shandong Province Statistical Yearbook (2017, 2022).”

### 2.4 Data analysis

Focusing on the price, availability and affordability of drugs, the World Health Organization (WHO) and the International Health Action Organization (HAI) have formulated the selection methods for investigating drugs and institutions, and determined the evaluation methods such as the median price ratio, the availability rate, and the number of days when the treatment cost of a single course of drugs is equivalent to the minimum wage of government unskilled workers. This study mainly refers to standard WHO/HAI methodology to analyze the price, consumption and affordability of anticancer drugs in Shandong Province. This method is scientific and reliable after professional research and verification. At present, this method has been applied in the study of drug availability and affordability in many countries and regions (22).

#### 2.4.1 Price

Following the WHO/HAI methodology, medicine prices were compared with international reference prices (IRPs) to obtain a median price ratio (MPR). MPR is an important indicator to evaluate the price level of medicines in the survey area, which is an expression of how much greater or less the local medicine price is compared to the IRP (23). The MPR is calculated as follows (24):


$$\mathrm{Median Price Ratio}\;\left(MPR\right)=\frac{\mathrm{median local unit price}}{\mathrm{international reference unit price}}$$

MPR = 1 is usually set as the threshold value, and if MPR < 1, it means that the price of the drug in China is lower than the International Reference Price (IRP). The WHO/HAI methodology recommends using the International Medical Products Price Guide published by the Management Science for Health (MSH) as the International Reference Price (IRP). The methodology also points out that if the research needs to choose another set of reference prices, it can consider the price of New Zealand Drug Administration (PHARMACY) or the price of Australian Drug Welfare Program (PBS). In this study, as only a single anticancer drug was featured in the International Medical Products Price Guide, we consistently utilized the price of this drug listed in the Pharmaceutical Benefits Scheme (PBS) as the international reference price (25, 26). In addition, since our prices came from the hospitals and were not reimbursement prices, we adopted the “dispensed price for maximum quantity” (DPMQ) of PBS as reference prices. At the same time, the conversion between RMB and Australian dollar was also carried out. Purchasing Power Parity (PPP) can more aptly reflect the actual difference between the purchasing power of the two countries, which is less volatile and relatively stable, so this study chooses the PPP value for conversion (27). In this study, the PPP conversion factor of private consumption calculated by the World Bank was used to convert the RMB price and PSB of anticancer drugs according to the purchasing power parity conversion factor, and the conversion calculation method was the same as the exchange rate conversion method. Since the pre-negotiation prices are for 2018 and 2019 drugs and the post-negotiation prices are for 2022 prices, which also need to be taken into account in calculating the MPR through inflation/deflation, we have adjusted all prices uniformly to 2023 levels. Referring to the method of drug price conversion provided by the WHO, we used the Consumer Price Index (CPI) as the conversion factor to adjust the prices uniformly. According to China’s National Bureau of Statistics (NBS), the consumer price index (CPI) is 102 in 2022 (compared to 100 in the previous year) (28).

Formula of inflation factor: $\mathrm{IF}=\left[1+\left(\frac{{CPI}_{i}-{CPI}_{j}}{{CPI}_{i}}\right)\right]$ , I and j denote different years, and the inflation factor for year i relative to year j.

Formula of price conversion: $P_{i}=P_{j}\times \mathrm{IF}\text{.}$

#### 2.4.2 Laspeyres Price Index

The Laspeyres Price Index was put forward by German economist Etienne Laspeyres in 1864 (29). The index is calculated as the ratio of the current price to the base period price using the base period quantity (Q0) as the weight, which can reflect the change of the drug price fixed in the base period. The calculation formula is as follows:


$$L_{p}=\sqrt{\frac{\sum P_{1}Q_{0}}{\sum P_{0}Q_{0}}}$$

#### 2.4.3 Usage

Expressed as the Defined Daily Doses (DDDs) of drug use, larger DDDs indicate a greater tendency for the usage of the drug in practice, the calculation formula of DDDs is:


$$\mathrm{Defined Daily Doses}\;\left(\mathrm{DDDs}\right)=\frac{\mathrm{Total drug dosage}}{DDD\;\mathrm{value of the drug}}$$

Defined Daily Dose (DDD) values were obtained from the official website of the WHO Collaborating Center for Drug Statistics Methodology (WHOCC) (30); for drugs without a DDD value assigned by the WHOCC, the usual daily dose for adults as specified by the National Medical Products Administration (NMPA) in the drug’s instructions will prevail. Moreover, anticancer medicines administered based on the patient’s weight or surface area are partially calculated assuming a reference adult weight of 70 kg and a body surface area of 1.7 m^2^ (31).

#### 2.4.4 Affordability

The affordability assessment in the WHO/HAI standardized approach was based on the minimum daily wage of an un-skilled government worker (LPGW) and calculates the number of days that the cost of a prescribed course of treatment for a drug for a disease is equivalent to the minimum daily wage of an LPGW. Since there is a lack of official data on the LPGW minimum daily wage in China, this paper uses disposable income per person per day for urban and rural residents as an alternative (32, 33). Based on available studies, affordability in this paper was calculated as the number of days of disposable income per capita needed to cover the cost of a one-month (30-day) course of each anticancer medicine (34, 35). A drug is considered affordable when the cost of the one-month drug course is less than the average daily per capita disposable income of urban and rural residents. Since anticancer medicines are costly and require long-term treatment, so that the affordability results calculated in this way all exceed 1 day’s disposable income per capita. Therefore, this study also incorporates Rasha Khatib et al.’s method: a month of treatment is considered affordable if it costs less than 20% of the household’s ability to pay (36), which has been applied in previous studies (37, 38).

Besides, as urban residents have higher incomes than rural residents, this study collected data on per capita disposable income and average monthly household income separately for each category. Since we used the per capita disposable income of urban and rural residents, only the basic medical insurance for urban and rural residents was considered. The design and benefits of basic medical insurance for urban and rural residents may vary among cities in Shandong Province, so we use the average reimbursement rate for hospital costs as a substitute to estimate the actual affordability level of anticancer medicines in Shandong Province.

In 2022, the average reimbursement ratio of hospitalization expenses for basic medical insurance for urban and rural residents reached 65.90% (39). Since the reimbursement categories of all 25 medicines included in the study belong to Medicare Category B medicines (medicines that are available for clinical treatment options, have good efficacy, and are more expensive than Category A medicines in their list). Shandong Provincial Healthcare Security Administration stipulates that the proportion of patients who need to pay out-of-pocket for Medicare Category B medicines should not exceed 20% in principle (40), from the current documents issued by various local cities, the ratio is between 10 and 20%. In summary, this paper set a 15% individual out-of-pocket payment ratio for patients, and then calculated the cost of drugs at a reimbursement rate of 65.90%.

SPSS 25.0 was used for statistical analysis and non-parametric test (Mann–Whitney U test) was used to analyze whether there was a difference in MPR values of medicines before and after negotiation, and descriptive statistics were used to summarize the characteristics of DDDs and affordability, with the statistical significance level set at *p* < 0.05.

## 3 Results

### 3.1 Price

#### 3.1.1 Price changes of 25 drugs before and after negotiations

Since the NHPA stipulates that the drugs negotiated in 2018 and 2019 have to be made public on the centralized drug procurement platform by the end of October 2018 and by the end of December 2019, respectively. Therefore, the pre-negotiation period for drugs successfully negotiated in 2018 was set as October 2017 to October 2018. The pre-negotiation period for 2019 is set for the current year, and the price after negotiation will be based on the drug price in 2022.

Prior to the negotiations, the unit prices of 25 anticancer drugs ranged from CNY 294.93 (USD 73.91) to CNY 18,877.25 (USD 4,638.37). After negotiations, the price ranged from CNY 53.79 (USD 13.48) to CNY 7,224.52 (USD 1,810.66). The median price of drugs before and after the negotiations was CNY 1725.49 (USD 432.45) and CNY 196.84 (USD 49.33) respectively, with a significant reduction in drug prices. After negotiations, there are still six anticancer medicines priced at more than CNY 1,000. Respectively Octreotide 20 mg/30 mg (CNY 5301.69/ USD 1328.74、CNY 7224.52/ USD 1810.66), Pertuzumab (CNY 4960.28/ USD 1243.18), Ixazomib 3 mg/4 mg (CNY 3978.14/ USD 997.03、CNY 4937.15/ USD 1237.38), Pegaspargase (CNY 2982.20/ USD 747.42), Cetuximab (CNY 1166.03/ USD 292.24) and Sintilimab (CNY 1081.03/ USD 270.93). See Table 2 for details.

**Table 2:** General information and MPR of 25 anticancer medicines.

Generic name	Dosage form	Drug Specifications	Median Unit price(CNY)		MPR	
			Pre-negotiation	Post-negotiation	Pre-negotiation	Post-negotiation
Afatinib	Tablets	30 mg	NA	160.66	NA	0.32
		40 mg	NA	200.22	NA	0.40
Alectinib	Capsules	150 mg	NA	63.30	NA	0.11
Axitinib	Tablets	5 mg	NA	196.84	NA	0.23
Azacitidine	Injections	100 mg	NA	950.13	NA	0.35
Anlotinib	Capsules	8 mg	NA	215.17	NA	NA
		10 mg	NA	255.26	NA	NA
		12 mg	NA	293.50	NA	NA
Olaparib	Tablets	150 mg	NA	102.10	NA	0.30
Octreotide	Microsphere injections	20 mg	9649.00	5301.69	2.96	1.66
		30 mg	10770.00	7224.52	2.89	1.98
Osimertinib	Tablets	80 mg	1760.00	186.19	2.23	0.24
Pyrotinib	Tablets	80 mg	NA	71.45	NA	NA
Fruquintinib	Capsules	1 mg	NA	89.87	NA	NA
		5 mg	NA	359.69	NA	NA
Crizotinib	Capsules	200 mg	NA	192.97	NA	0.57
		250 mg	890.42	229.03	2.60	0.68
Raltitrexed	Injections	2 mg	1504.98	666.63	0.45	0.20
Ruxolitinib	Tablets	5 mg	112.67	53.79	NA	NA
Nilotinib	Capsules	150 mg	NA	73.82	NA	0.79
		200 mg	300.83	92.02	2.40	0.75
Pertuzumab	Injections	420 mg:14 mL	18877.25	4960.28	1.07	0.29
Pegaspargas	Injections	2 mL:1500 IU	3570.00	2982.20	NA	NA
Pazopanib	Tablets	200 mg	NA	160.16	NA	1.54
Regorafenib	Tablets	40 mg	306.00	172.65	NA	NA
Ceritinib	Capsules	150 mg	NA	136.14	NA	0.34
Sunitinib	Capsules	12.5 mg	NA	155.16	NA	1.85
Vemurafenib	Tablets	240 mg	NA	89.77	NA	0.26
Cetuximab	Injections	20 mL:100 mg	3463.17	1166.03	0.69	0.24
Sintilimab	Injections	10 mL:100 mg	7870.17	1081.03	NA	NA
Ibrutinib	Capsules	140 mg	540.00	169.17	1.97	0.63
Ixazomib	Capsules	3 mg	NA	3978.14	NA	NA
		4 mg	NA	4937.15	NA	NA

NA, Not available.

The MPRs of the included drugs were obtained by comparing the prices of nationally negotiated anticancer drugs with IRP. The MPR range of the 8 anticancer medicines before the negotiation was 0.45–2.96, with a median of 2.23. Six anticancer medicines are priced significantly higher than international reference prices, after negotiations, the MPR range is 0.19–1.98, with a median of 0.57, among them, 17 medicines have MPR <1, accounting for 80.95%, and 4 drugs have MPR >1, accounting for 19.05%. The Mann–Whitney U test yielded that there was a significant difference in the MPR values before and after negotiation, and the post-negotiation price was significantly lower than the pre-negotiation price (*p* < 0.01).

#### 3.1.2 Changes in the price index of drugs in different negotiation batches

**Figure 1:**
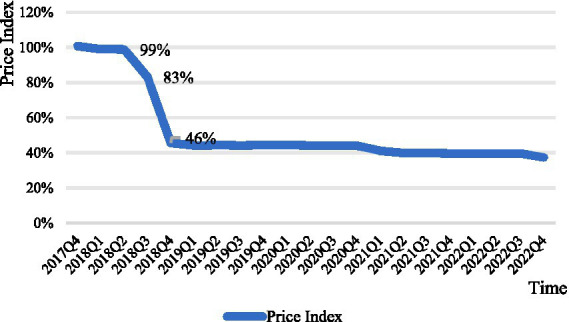
Negotiated anticancer medicines price index 2018.

**Figure 2:**
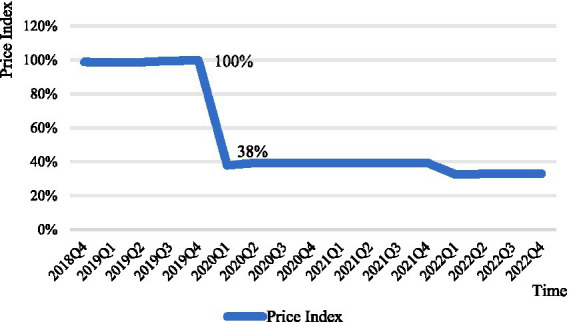
Negotiated anticancer medicines price index 2019.

Overall, the price index curves for anticancer drugs negotiated in both 2018 and 2019 show a substantial decline, stabilizing around the time of the decline. The negotiation time for anticancer drugs in 2018 and the time it was stipulated to be included in the medical insurance catalog are June and October 2018, respectively. As shown in Figure 1, the drug price index showed a significant decline during this period. The negotiation time for anticancer drugs in 2019 and the time it was stipulated to be included in the medical insurance catalog are November and December 2019, respectively. It can be seen that the price index curve almost experienced a sharp decline in that quarter, with the post-negotiation price index falling by 62% compared to the pre-negotiation period (Figure 2).

### 3.2 Usage

#### 3.2.1 Use of anticancer medicines for different indications

**Figure 3:**
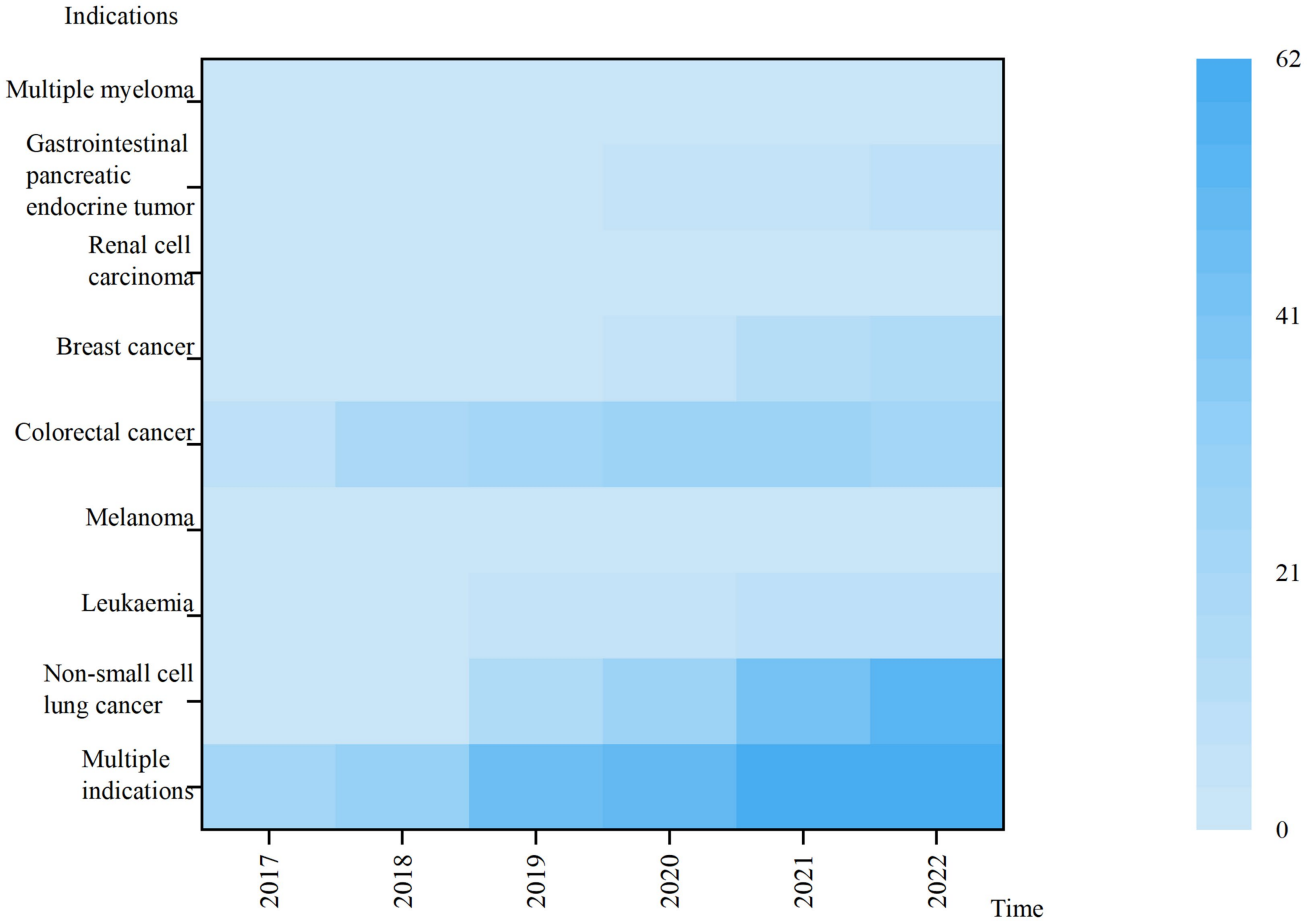
Heat map of anticancer medicines usage for different indications.

From 2017 to 2022, the highest total usage was for multi-indication anticancer drugs. Lower use of drugs with indications of Melanoma, Multiple myeloma, Renal cell carcinoma, Gastrointestinal pancreatic endocrine tumor and leukemia, the usage of the above five categories accounted for less than 5% of the total usage in 2022. The use of drugs under all indications has shown a year-on-year increase, with large variations in the use of anticancer drugs under different indications (Figure 3).

#### 3.2.2 Changes in the use of 25 anticancer medicines

This study was conducted to analyze the use of anticancer drugs based on the hospital medicine usage from 2017 to 2022. Among the 25 kinds of anticancer drugs, the usage of 15 kinds of drugs showed a continuous rising trend, among which the highest average annual growth rate was 1011.26% for Sintilimab and the smallest was 8.20% for Pegaspargas. The use of 10 other drugs showed a trend of increasing and then decreasing, the largest decrease from 2021 to 2022 is 44.72% for Crizotinib and the smallest is 0.91% for Afatinib. Raltitrexed is the only one of the 25 anticancer drugs whose use continued to decline after the negotiations, with a 19.05% reduction.

**Figure 4:**
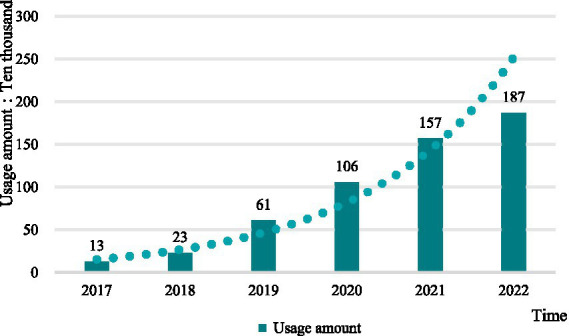
Total use of anticancer medicines by year.

From the perspective of the total drug use, after the drug price negotiation, the total amount of anticancer drugs used in hospitals in Shandong Province showed a continuous upward trend, with an average annual growth rate of 59.62% in 6 years, but the growth rate of drug use in 2022 was slower than that in 2020 and 2021 (Figure 4).

### 3.3 Affordability

Table 3 details the number of days of disposable income per capita needed to cover 1 month’s drug costs for 25 nationally negotiated anticancer drugs. Before the negotiations, the monthly treatment costs of anticancer drugs were equivalent to the per capita disposable income of urban residents for 20 (Raltitrexed) to 233 (Crizotinib) days, respectively and 48 (Raltitrexed) to 567 (Crizotinib) days of disposable income per capita for rural residents. The average affordability for urban and rural residents is 104 and 256 days of per capita disposable income, respectively.

**Table 3:** Usage of 25 types of anticancer medicines (mg).

Generic name	2017	2018	2019	2020	2021	2022
Afatinib	NA	474.25	18104.75	33291.75	46109.00	45689.00
Alectinib	NA	NA	NA	10276.00	19287.00	19736.75
Axitinib	70.00	224.00	6669.00	9759.70	15091.60	20679.00
Azacitidine	NA	2438.22	106342.67	239341.02	349456.34	467413.51
Anlotinib	NA	1264.67	74375.67	104346.33	142122.50	136842.33
Olaparib	NA	NA	NA	6051.75	12497.25	17108.00
Octreotide	1676.06	2028.57	15628.57	36528.57	64171.43	69585.71
Osimertinib	30.00	1170.00	53168.00	90154.00	215743.00	335604.00
Pyrotinib	NA	NA	NA	7968.40	14836.60	15279.80
Fruquintinib	NA	NA	NA	2838.40	10382.60	11528.20
Crizotinib	120.00	570.00	14664.00	16024.50	16374.50	9052.60
Raltitrexed	100975.00	193275.00	209741.67	236308.33	233333.33	191300.00
Ruxolitinib	NA	NA	20.00	7118.00	10663.67	13053.33
Nilotinib	640.00	1040.00	15664.00	24942.83	34541.08	34079.58
Pertuzumab	NA	NA	462.00	53676.00	113946.00	120897.00
Pegaspargas(IU)	19801.86	20258.92	24261.29	28461.31	29140.73	29375.43
Pazopanib	NA	75.00	2402.50	8621.75	9820.50	10309.50
Regorafenib	NA	298.67	15641.33	24635.67	29614.33	32998.00
Ceritinib	NA	166.67	1439.00	2558.33	3062.33	7731.67
Sunitinib	238.00	84.00	8259.00	12197.00	13108.75	11854.25
Vemurafenib	NA	56.00	1040.13	913.38	702.88	351.50
Cetuximab	3989.46	2706.31	23450.83	25121.07	32637.13	14786.69
Sintilimab	NA	NA	168.07	55661.76	112815.13	230640.76
Ibrutinib	NA	630.00	11454.50	14268.00	16718.50	9579.25
Ixazomib	NA	242.11	6819.30	13011.93	22617.54	17891.23

After the negotiations, the cost of a month’s worth of drugs is calculated by using the resident’s personal out-of-pocket payment of 15% and reimbursement of the remaining costs at 65.90%. Affordability ranged from 6 (Azacitidine) to 77 days (Axitinib) for urban residents and 15 (Azacitidine) to 188 days (Axitinib) for rural residents. The average affordability for urban and rural residents is 36 and 88 days of per capita disposable income, respectively. Based on the monthly cost of drugs as 20% of average monthly household income, three drugs were affordable to urban residents, accounting for 12.00% of the total number of drugs studied. Rural residents can afford only one drug (Tables 4, 5).

**Table 4:** Affordable days for patients with 25 anticancer medicines.

Generic name	DDDc	Total usage per month	Health insurance catalog	Pre-negotiation		Post-negotiation	
				Urban residents	Rural residents	Urban residents	Rural residents
Afatinib	40 mg	1,200 mg	Class B	NA	NA	20	48
Alectinib	1,200 mg	27,600 mg	Class B	NA	NA	38	93
Axitinib	10 mg	600 mg	Class B	NA	NA	77	188
Azacitidine	6.07 mg	170 mg	Class B	NA	NA	6	15
Anlotinib	12 mg	276 mg	Class B	NA	NA	22	54
Olaparib	600 mg	18,000 mg	Class B	NA	NA	40	98
Octreotide	0.7 mg	21 mg	Class B	31	77	17	42
Osimertinib	80 mg	2,400 mg	Class B	172	421	18	45
Pyrotinib	400 mg	12,000 mg	Class B	NA	NA	35	85
Fruquintinib	5 mg	115 mg	Class B	NA	NA	27	66
Crizotinib	500 mg	15,000 mg	Class B	174	426	45	110
Raltitrexed	0.24 mg	5.1 mg	Class B	15	36	7	16
Ruxolitinib	30 mg	900 mg	Class B	66	162	32	77
Nilotinib	600 mg	18,000 mg	Class B	88	216	27	66
Pertuzumab	20 mg	420 mg	Class B	61	151	16	40
Pegaspargas	303.57 IU	8,500 IU	Class B	23	57	19	48
Pazopanib	800 mg	24,000 mg	Class B	NA	NA	63	153
Regorafenib	120 mg	2,760 mg	Class B	69	168	39	95
Ceritinib	450 mg	13,500 mg	Class B	NA	NA	40	98
Sunitinib	50 mg	1,400 mg	Class B	NA	NA	57	139
Vemurafenib	1920 mg	57,600 mg	Class B	NA	NA	70	172
Cetuximab	60.71 mg	1700 mg	Class B	NA	NA	65	158
Sintilimab	9.52 mg	200 mg	Class B	NA	NA	7	17
Ibrutinib	560 mg	16,800 mg	Class B	NA	NA	66	162
Ixazomib	0.43 mg	12 mg	Class B	NA	NA	48	118
				104	254	36	88

**Table 5:** Proportion of monthly drug costs exceeding 20% of average monthly household income.

	Pre-negotiation		Post-negotiation	
	Urban residents	Rural residents	Urban residents	Rural residents
Number (percentage)	25 (100%)	25 (100%)	22 (88%)	24 (96%)

## 4 Discussion

In this study, the MPR was used to analyze the prices of nationally negotiated anticancer drugs in Shandong Province. Compared to 2017, the MPR range for 25 anticancer drugs in 2022 is 0.19–1.98, with a median decrease from 2.23 to 0.57, anticancer drug prices are mostly lower than IRPs. The NHSA released information that the prices of drugs that passed negotiations in 2018 and 2019 were reduced by an average of 56.7 and 60.7%, respectively, compared with the pre-negotiation market retail prices (41), which is consistent with the results of this study. It shows that the drug price negotiation policy organized by China’s NHSA has achieved good results. A survey in Nanjing City found that after negotiations, the price reduction rate of innovative anticancer drugs was between 34 and 65% (17), another study counted the price reductions in the first five batches of negotiated drugs and found that all five batches had price reductions of more than 44 percent (42). For cancer patients, lower prices of anticancer drugs can appropriately reduce the financial pressure on families, avoid treatment interruptions or changes in treatment programs for financial reasons.

From 2017 to 2021, the use of all drugs except Retitraxel increased continuously, but by 2022, the use of 10 drugs declined, and the growth rate of the total use of anticancer drugs in negotiations slowed down significantly, which was the lowest year since the negotiations. The incidence of cancer is on the rise, the price of anticancer drugs is falling, and the number of drugs in the catalog is increasing, while the growth rate of drug use in hospitals is slowing down instead. This may be caused by the influence of the “dual channel” policy.

In May 2021, in order to meet the needs of patients and improve the availability of negotiated drugs, the NHSA issued guidelines for establishing and improving the “Dual Channel” management of nationally negotiated drugs (43). The “Dual Channel” refers to two channels: designated medical institutions and designated retail pharmacies. The NHSA clearly stipulates those drugs with high clinical value, urgent needs of patients and low availability among the negotiated drugs will be included in the “Dual Channel” management in a timely manner, i.e., such drugs are subject to uniform payment standards in medical institutions and pharmacies. We should give full play to the advantages of widely distributed retail pharmacies and flexible services, and complement each other with designated medical institutions to better ensure the supply of negotiated drugs. Therefore, changes in drug utilization in 2022 may be related to the replenishment of sentinel retail pharmacies. In a study in Guizhou Province, China, a significant increase in prescription volume was found after the implementation of “Dual Channel” management, among them, the prescriptions transferred out of hospital accounted for 56.53% of the total prescriptions (44).

In terms of affordability of drugs, prior to the negotiations, the average affordability of urban and rural residents was 78 and 190 days of per capita disposable income required for monthly treatment costs, respectively. After the negotiations, the average affordability for urban and rural residents is 36 and 88 days of disposable income per capita, respectively. The NDPN policy has significantly improved the affordability of drugs for urban and rural residents. Previous studies have had similar results, in a survey of 29 provinces in mainland China, it was found that the daily cost of patients using the first negotiated cancer drug dropped by more than 50 percent (14), in 2018, the cumulative reimbursement amount of 17 anticancer medicines in 5 sample cities reached 378 million yuan (45), which improved financial pressure on patients and increased affordability of medicines.

However, the monthly treatment costs were still found far exceeded the per capita disposable income for one day. Since innovative anticancer medicines were originally priced at a high level, so the substantial increase in the average affordability of urban and rural residents after the drug price reduction can illustrate the superiority of the NDPN, particularly instrumental in reducing drug prices. However, based on the cost of drugs at 20% of average monthly household income, after the negotiation, urban residents can afford three medications and rural residents can only afford one, which shows that anticancer medicines are more likely to bring economic burden than other diseases. About half of all cancer patients in China have borrowed money or are in debt due to their illness, and younger cancer survivors experience more severe material and financial difficulties compared to older patients (46).

The reason for this phenomenon may be due to the high royalties of most of the negotiated medications and the existence of certain tariffs and VAT on the introduction of foreign drugs into the country. Furthermore, China has a large population with a high incidence of malignant tumors and a high demand for anticancer drugs, the combination of the market price theory and the monopolistic nature of the manufacturers makes the prices of drugs is still high even after negotiation (47). It is also possible that the negotiations were not strong enough because the government did not have enough access to information before the negotiations or there were fewer manufacturers participating in the bidding to accurately predict the actual cost of drugs (48). Therefore, it is necessary to encourage domestic manufacturers to develop new drugs or high-quality generic drugs to form an effective market competition, using the market to regulate drug prices is an effective way to reduce the burden of drug use on patients, and also to develop incentive mechanisms for anticancer medicine R&D and actively promote the review of innovative anticancer drugs and generic drugs.

The financial burden associated with the use of anticancer drugs is not only the price factor of the drugs themselves, but also the treatment time and social factors that cannot be ignored. Unlike most chronic diseases, oncology diseases have long treatment cycles and patients often need to take medication for a long time or even for life. Therefore, even if the price of drugs drops significantly after negotiations, most patients are still prone to financial problems after a long period of medicine use. In addition, some patients are incapacitated because of their illness. Occurrence of poverty due to illness, it can further exacerbate the disease burden. Among the BRICS countries, China’s productivity loss due to premature deaths due to cancer stands at $28 billion, the highest among the five countries (49).

Furthermore, a large difference was found in the average affordability of urban and rural residents in Shandong Province, which may be related to the large income gap between urban and rural residents. Taking the 2022 Shandong Province data as an example, the per capita disposable income of urban residents is more than twice that of rural residents. Not only that, due to the unbalanced socioeconomic development between urban and rural areas in China, the socioeconomic status of middle-aged and older adult people in rural areas is more vulnerable than in urban areas, and the high medical expenses are more likely to impoverish them (50). Although the policy of universal coverage of basic medical insurance was implemented in China in 2016, there are still large disparities in medical insurance reimbursement ratios, medical resource allocation, and medical service coverage between different regions and groups, leading to the differentiation of urban and rural medical supply (51).

This study has several limitations: First, since the international drug price guide published by MSH includes only one medication in this study, the Australian PBS price was used as a reference. Australia is a developed country with a high level of economic development, its overall drug price level may be higher than that of China. MPR results from comparisons made in this context may be lower than actual, affecting the accuracy of price estimates. Second, the implementation time of the policy is short, and the data available is small, and only 25 drugs are included by the study date. In addition, for the drugs negotiated by the state, most of them are newly listed innovative drugs or exclusive products, which are different from the price characteristics of commonly used drugs in the market. Limited by the data source, this study did not test the same outcomes but for drugs that were not part of the national drug price negotiations to provide natural comparative. Despite these limitations, the research data in this paper can show that NDPN have effectively lowered drug prices, substantially increased affordability for urban and rural residents, and reduced the economic burden on patients.

## 5 Conclusion

After the NDPN, the prices of anticancer medicines dropped significantly, and most of them were lower than the IRPs. The use of negotiated drugs in hospitals has been increasing year by year, but at a slower rate. The average affordability of anticancer drugs for both urban and rural residents in Shandong Province increased significantly. However, there are large differences in burden levels between urban and rural residents. After the price reduction and reimbursement, the number of days per capita disposable income required for a month’s drug costs was still higher. Therefore, targeted measures should be taken to further improve the affordability of urban and rural residents. First, it is recommended that the research and development of quality generic drugs be encouraged to create reasonable market competition. Second, strengthen government negotiation efforts and conduct market surveys prior to negotiations. Lastly, the allocation of medical resources in rural areas should be optimized, employment should be improved and the income of rural residents should be raised, and assistance to rural patients and poor patients should be strengthened by means of diversified financing. The NDPN policy and the basic medical insurance system have a positive effect, but are insufficient to alter the affordability of anticancer drugs. In the future, efforts should be made to improve the multi-level medical insurance system for cancer patients. More cancer patients can rely on commercial insurance, medical assistance and other financing channels to afford anti-cancer drugs. Commercial medical insurance should be better used as a supplement to the basic plan to improve the coverage of anticancer drugs.

## Data Availability

The raw data supporting the conclusions of this article will be made available by the authors, without undue reservation.
